# Upregulation of Tolerogenic Pathways by the Hydrogen Sulfide Donor GYY4137 and Impaired Expression of H_2_S-Producing Enzymes in Multiple Sclerosis

**DOI:** 10.3390/antiox9070608

**Published:** 2020-07-10

**Authors:** Milica Lazarević, Giuseppe Battaglia, Bojan Jevtić, Neda Djedovic, Valeria Bruno, Eugenio Cavalli, Đorđe Miljković, Ferdinando Nicoletti, Miljana Momčilović, Paolo Fagone

**Affiliations:** 1 Department of Immunology, Institute for Biological Research “Siniša Stanković”—National Institute of Republic of Serbia, University of Belgrade, Despota Stefana 142, 11060 Belgrade, Serbia; milica.laza93@gmail.com (M.L.); bojanbh@gmail.com (B.J.); ndjedovic@yahoo.com (N.D.); georgije_zw@yahoo.com (Đ.M.); mommilja@yahoo.com (M.M.); 2 Department of Physiology and Pharmacology, Sapienza University, Piazzale A. Moro, 5, 00185 Rome, Italy; giuseppe.battaglia@uniroma1.it (G.B.); valeria.bruno@uniroma1.it (V.B.); 3 IRCCS Neuromed, Località Camerelle, 86077 Pozzilli, Italy; 4 Department of Biomedical and Biotechnological Sciences, University of Catania, Via S. Sofia 89, 95123 Catania, Italy; eugeniocavalli9@hotmail.it (E.C.); paolofagone@yahoo.it (P.F.)

**Keywords:** carbon monoxide, dendritic cells, experimental autoimmune encephalomyelitis, hydrogen sulfide, multiple sclerosis, nitric oxide, regulatory T cells, T cells

## Abstract

The aim of this study was to examine the in vitro effects of the slow-releasing H_2_S donor GYY4137 on the immune cells involved in the pathogenesis of the central nervous system (CNS) autoimmune disease, multiple sclerosis (MS). GYY4137 specifically potentiated TGF-β expression and production in dendritic cells and significantly reduced IFN-γ and IL-17 production in the lymph node and spinal cord T cells obtained from mice immunized with CNS antigens. Both the proportion of FoxP3+ regulatory CD4+ T cells in the lymph node cells, and the percentage of IL-17^+^ CD4+ T cells in the spinal cord cells were reduced upon culturing with GYY4137. Interestingly, the peripheral blood mononuclear cells obtained from the MS patients had a lower expression of the H_2_S-producing enzyme, 3-mercaptopyruvate-sulfurtransferase (MPST), in comparison to those obtained from healthy donors. A significant inverse correlation between the expression of MPST and several pro-inflammatory factors was also observed. Further studies on the relevance of the observed results for the pathogenesis and therapy of MS are warranted.

## 1. Introduction

Experimental autoimmune encephalomyelitis (EAE) is an animal model of the human chronic demyelinating inflammatory central nervous system (CNS) disease, multiple sclerosis (MS) [1,2]. The activation of naïve autoreactive CD4+ T cells by the recognition of CNS antigens in lymph nodes is the initial step of active EAE pathogenesis [3]. This step is enabled by dendritic cells (DCs), antigen-presenting cells (APCs) that are able of activating naïve T cells [4]. Prior to migration to the CNS, activated CD4+ T cells proliferate and differentiate towards the two main encephalitogenic T cell populations T helper (Th)1 and Th17 [5]. These cells migrate into the CNS, where they are re-activated by the local APCs [3,6]. The re-activation induces the inflammatory cascade, involving other immune cells, such as macrophages/microglia, B cells and neutrophils that ultimately cause tissue injury [3,6]. EAE and MS share several pathogenic mechanisms, including the major role of Th1 and Th17 CNS-reactive cells in the initiation of the tissue destruction [7]. Regulatory T cells (Treg) have the critical role in antagonizing CNS-reactive Th1 and Th17 cells in multiple sclerosis and EAE and the dysfunctions of Treg are considered as predisposing factors in the initiation and progression of the disease [7,8].

Together with nitric oxide (NO) and carbon monoxide (CO), H_2_S represents the third component of the family of endogenous gases that are involved in the maintenance of homeostasis [9,10]. A growing body of evidence suggests that the impairment of the metabolism of these gases contributes to the pathogenesis of several different diseases, including cancer, neuroinflammatory and neurodegenerative diseases [10,11,12,13].

The dismantling of the pleiotropic role of these gases in multiple biological processes has served not only as a theoretical basis for the better understanding of their key role in physiopathology, but it has also suggested the potential clinical translation of these concepts, as the modulation of the levels of these gases can be envisaged in a tailored fashion. For instance, we have previously shown that a CO-releasing molecule ameliorated the course of murine EAE [14] and we subsequently demonstrated that hem oxygenase 1 is reduced in the peripheral blood mononuclear cells (PBMCs) of MS patients, correlating with the clinical activity of the disease [15], thus validating the preclinical findings, and pointing out to a possible use of CO donors for the treatment of multiple sclerosis [16].

The possibility of modulating the eventually disregulated production of these three endogenous gases can be achieved by inhalation or by systemically administered donors or hybrids that consist of parental drugs linked to a moiety of one or more of these gases. In particular, we and others have shown that it is possible to generate NO and H_2_S hybrid molecules of parental compound, including the HIV antiretroviral protease inhibitors, nonsteroidal anti-inflammatory drugs and histone deacetylase inhibitors. These hybrid compounds acquire stronger pharmacological properties than their parental compounds, and often with the reduction of side effects [17,18,19,20,21,22,23,24,25,26,27]. More recent data indicate the possibility to generate double hybrids of H_2_S and NO-donating compounds that entail an even more complex and tailored mechanism of action than the parental drugs [28,29].

It is of relevance, for the purpose of this study, that multiple evidences have indicated the remarkable pharmacological properties of H_2_S in the setting of cancer, immunoinflammation and neurodegeneration [19,30,31,32,33,34]. H_2_S is ubiquitously present in mammalian tissues and organs in the nanomolar range and endogenously produced from cysteine by direct desulfhydration catalyzed by two pyridoxal-5’-phosphate-dependent enzymes: cystathionine β-synthase (CBS) and cystathionine γ-lyase (CTH), and by indirect desulfhydration catalyzed by 3-mercaptopyruvate-sulfurtransferase (MPST) [35,36]. A growing body of evidence suggests that the impairment of the H_2_S metabolism contributes to several neuroinflammatory and neurodegenerative diseases, including multiple sclerosis [11,12,13]. In line with that, it has been proposed that H_2_S application could ameliorate CNS disorders, via its effects on neurons, microglia and astrocytes [37,38,39]. Several studies have shown the anti-inflammatory effects of H_2_S, both in vitro and in vivo [40,41,42]. Nevertheless, pro-inflammatory effects have also been attributed to this gaseous molecule [43,44]. The research of the physiological and pharmacological roles of H_2_S largely relies on its donors, such as commonly used sulfide salts, NaSH and Na_2_S [45]. GYY4137 was increasingly recognized as a useful compound for elucidating the biological roles of H_2_S [45,46]. GYY4137 is a derivative of Lawesson’s reagent and releases H_2_S via hydrolysis [45,46], as well as a slow-releasing donor of H_2_S, as the release of H_2_S from GYY4137 is measured by minutes and hours, in comparison to sulfide salts which release H_2_S within seconds [45].

In our previous paper, we have shown that GYY4137 down-regulated TNF and NO secretion, as well as CD40 and CD86 co-stimulatory molecule expression, in microglial cells [47]. In this study, we extended our area of research in MS to the effect of GYY4137 on DCs and T cells that are involved in the initiation and maintaining of the pathogenesis of MS and EAE. Moreover, using in silico methods, we examined the expression of three H_2_S-producing enzymes in the immune cells from healthy donors and MS patients.

Our results imply that GYY4137 specifically potentiates the transforming growth factor (TGF)- expression and production in dendritic cells. Moreover, this H_2_S donor significantly reduces interferon (IFN)-γ and interleukin (IL)-17 production in the lymph node and spinal cord encephalitogenic T cells. Moreover, the lower levels of MPST were detected in the PBMCS of MS patients as compared to those in healthy donors’ PBMCs. Interestingly, MPST expression was inversely correlated to several pro-inflammatory factors.

## 2. Materials and Methods

### 2.1. In Vitro, In Vivo and Ex Vivo Studies

#### 2.1.1. Cells and Cell Cultures

Dark Agouti rats or C57BL/6 mice were used for the isolation of the cells (experiments were approved by the local ethics committee of the Institute for Biological Research “Siniša Stanković”, N°04-10/19 01-2276, in accordance with Directive 2010/63/EU). All the cell cultures were mantained at 37 °C, 5% CO_2_, hand under a humid atmosphere.

C57BL/6 mice femurs were used for the isolation of bone marrow cells for the differentiation of bone marrow-derived dendritic cells (BMDCs). For the cultivation of bone marrow cells, a RPMI-1640 medium (Biological Industries, Kibbutz Beit-Haemek, Israel) supplemented with 20% fetal calf serum (FCS, PAA Laboratories, Pasching, Austria), 2 mM glutamine and 1 mM sodium pyruvate (both from Sigma-Aldrich, St. Louis, IL, USA) was used. The cells were grown at 1 × 10^6^/mL/well in a 24-well plate, in the presence of 20 ng/mL of granulocyte-macrophage colony-stimulating factor (GM-CSF, Peprotech or Novus, Littleton, CO). Cultivation lasted for 8 days, while 100 ng/mL lipopolysaccharide (LPS, Sigma-Aldrich, St. Louis, USA), as the maturation stimuli, was applied on day 7 of cultivation. Treatment with 200 μM GYY4137 (Cayman Chemical, Ann Arbor, MI, USA) was performed simultaneously with LPS. GYY4137 was initially diluted in DMSO (Sigma-Aldrich) and DMSO was used as the vehicle control in all the experiments. In some experiments, GYY4137 was kept at 37 °C, 5% CO_2_ for 7 days before being used in cell cultures (spent GYY4137). Na_2_S (Sigma-Aldrich, St. Louis, USA) was applied at 200 μM.

Draining lymph node cells (DLNCs) were isolated by the mechanical disruption from draining (popliteal) lymph nodes of Dark Agouti rats and C57BL/6 mice, immunized with 0.5 mg/mL guinea pig myeline oligodendrocyte glycoprotein (MOG, obtained from Professor Alexander Flügel, University of Göttingen, Germany) mixed with an equal volume of complete Freund’s adjuvant (CFA, Difco, Detroit, MI, USA). The rats and mice were injected subcutaneously into hind hock with 100 μL or 20 μL of MOG + CFA, respectively. Draining lymph nodes were isolated on day 7 post-immunization. In all the experiments, the DLNCs were cultured in a RPMI-1640 medium, supplemented with 2% rat or mouse serum. For the purpose of the cytokine level measurement, the DLNCs were seeded at 5 × 10^6^/mL/well in a 24-well plate, stimulated with 15 μg/mL MOG and treated with 200 μM GYY4137. The measurement of the cytokine concentration in the cell culture supernatants was performed after 24 h by ELISA.

The spinal cord immune cells (SCICs) were isolated from the rats that were immunized with rat spinal cord homogenate (SCH) in phosphate buffer saline (PBS, 50% *w*/*v*), mixed with an equal volume of CFA, supplemented with 5mg/mL of *M. tuberculosis*. The rats were injected into hind hock with 100 μL SCH + CFA. Clinical signs of EAE were scored as follows: 0, no clinical signs; 1, flaccid tail; 2, hind limb paresis; 3, hind limb paralysis; 4, moribund state or death. After perfusion with cold saline, the spinal cords were isolated from the rats at the peak of the disease, on day 12 post-immunization (clinical score 2 or 3). The spinal cords were homogenized and centrifuged in 30/70% Percoll gradient (Sigma). Subsequently, the SCICs were recovered from the interface of the Percoll gradient and then washed in a RPMI medium. In all the experiments, the SCICs were cultured in a RPMI-1640 medium, supplemented with 5% FCS. For the purpose of the cytokine level measurement, the SCICs were seeded at 2.5 × 10^6^/mL/well in a 24-well plate and treated with 200 μM GYY4137. The measurement of the cytokine concentration in the cell culture supernatants was performed after 24 h by ELISA.

The CD4+ T cells were purified from mouse/rat DLNCs and SCICs by magnetic separation with biotin-conjugated antibody specific for rat CD4 (BD Pharmingen, San Jose, CA, USA) or mouse CD4 (Invitrogen, Carlsbad, CA, USA) and IMagSAv Particles Plus (BD Biosciences, San Diego, CA, USA).

In order to determine the percentage of Treg by cytofluorimetry, DLNCs, SCICs and CD4+ T cells were seeded at 1 × 10^6^/mL/tube in FACS (Fluorescence-Activated Cell Sorting) tubes and treated with 200 μM GYY4137 for 40 min, with no additional stimulation. In some experiments, mouse DLNCs were cultivated in the presence of 200 μM Na_2_S or or in the presence of 200 μM GYY4137, that was previously kept in a cell culture medium at 37 °C for 7 days (spent GYY4137). For the assessment of the kinetic of GYY4137 effects on Treg, the mouse DLNCs were cultured with GYY417 for 40 min, 1, 2, 3, 4, 5, 6 or 12 h. In order to determine the percentage of Th17 cells by cytofluorimetry, the DLNCs, SCICs and CD4+ T cells were seeded at 1 × 10^6^/mL/tube in FACS tubes and treated with 200 μM GYY4137 for 40 min, and subsequently stimulated with a cell-stimulation cocktail (eBioscience, San Diego, CA, USA), containing phorbol myristate acetate, ionomycin, brefeldin A and monensin, for 4 h, or, in some experiments, for 16 h. In the initial experiments, the cultures without DMSO (Ctrl) were performed to see if the DMSO itself had some effect.

#### 2.1.2. Cell Viability Assay

The viability of the BMDCs was assessed by 3-(4,5-dimethylthiazol-2-yl)-2,5-diphenyltetrazolium bromide (MTT) assay. The cells were collected in tubes, spun down and the supernatants were removed. The pellets were dissolved in 0.5 μg/mL MTT (Sigma-Aldrich) solution and incubated for 30 min at 37 °C to allow the mitochondria-dependent reduction of MTT to formazan. After 30 min, the cells were centrifuged and formed formazan crystals that were dissolved in DMSO. The number of viable cells was expressed as the absorbance of dissolved formazan. The absorbance was measured at 540 nm with a correction at 690 nm on an automated microplate reader (LKB 5060-006, LKB, Vienna, Austria).

#### 2.1.3. Cytokine Level Measurement

The cytokine concentration in cell culture supernatants was measured by sandwich ELISA. ELISA was performed in MaxiSorp plates (Nunc, Rochild, Denmark) with paired antibodies, according to the manufacturer’s instruction. The samples were analyzed in duplicates for murine TNF, murine IFN-γ, murine/rat IL-17 (all from eBioscience), murine IL-6 and rat IFN-γ (both from R&DSystems, Minneapolis, MN). For all the ELISA tests, the lower limit of detection was 30 pg/mL, while the upper limit of detection was 10 ng/mL. If the values over the upper limit were detected, the appropriate samples were adequately diluted for measurement. Absorbance was read using an automated microplate reader (LKB 5060-006). The results were calculated using standard curves, based on the absorbance of the known concentrations of recombinant cytokines.

#### 2.1.4. Cytometry

The cells were stained with phycoerythrin (PE)-conjugated anti-mouse MHC II (M5/114.15.2), fluorescein isothiocyanate (FITC)-conjugated anti-mouse CD40 (HM40-3), PECyanine5-conjugated anti-mouse CD40 (HM40-3), FITC-conjugated anti-mouse CD4 (RM4-5), PE-conjugated anti-mouse CD25 (PC61.5), FITC-conjugated anti-rat CD4 (OX35), PE-conjugated anti-rat CD25 (OX39), PE-cyanine5-conjugated anti-mouse/rat FoxP3 (FJK-16s), PE or PerCP-Cy5.5-conjugated anti-mouse/rat IL-17 antibodies (eBio17B7), (all from eBioscience). FoxP3 and IL-17 staining was performed according to the manufacturer’s instructions. Cells were stained with the appropriate isotype control antibodies in order to set the gates for marker positivity. In all cases, the proportion of the isotype control antibodies-stained cells was less than 1%.

Latex beads (1 μm, yellow-green, Sigma-Aldrich) were used for the detection of phagocytosis. The beads were opsonized in PBS supplemented with 50% FCS, and subsequently added to BMDC culture on the day 8 of cultivation (10 beads per cell) and incubated at 37 °C for an additional hour.

Dihydrorhodamine 123 (DHR, Sigma-Aldrich) staining was performed for the detection of ROS generation. The DCs were harvested on day 8 of cultivation, incubated in the presence of 5 μM DHR for 20 min and stimulated with 100 ng/mL phorbol 12-myristate 13-acetate (PMA, Sigma-Aldrich) for an additional 90 min.

The cells were analyzed using a CyFlow Space flow cytometer (Partec, Munster, Germany) (Supplementary Figures S1 and S2 show the gating strategy). Forward (FSC) and side (SSC) scatter were used to distinguish the live cells from the dead cells and debris. The FSC-w was used to exclude the doublets and multiplets from the analysis. The results are presented as the percentage of cells or mean fluorescence intensity (mfi) of the cell population.

#### 2.1.5. Reverse Transcription—Real-Time Polymerase Chain Reaction

Total RNA was isolated and RT-PCR was performed as described previously [46]. The PCR primers (Metabion, Martinsried, Germany) were as follows: TNF: 5′-CCA CGT AGC AAA CCA C-3′; 5′-TGG GTG AGG AGC ACG TAG T-3′; β-actin: 5′-CCA GCG CAG CGA TAT CG-3′; 5′-GCT TCT TTG CAG CTC CTT CGT-3′; iNOS: 5′-CTG CAG CAC TTG GAT CAG GA-3′; 5′-GCC AGA AAC TTG GGA AGG GA-3′; Arg1: 5′-CCT GCT GTC CTG TGA TAC CC-3′; 5′-CGG CTG TGC ATC ATA CAA CG-3′; IDO: 5′-TGG GCT TTG CTC TAC CAC AT-3′; 5′-GGC AGC ACC TTT CGA ACA TC-3′; IL-27: 5′-GCC AGG ACA CTT GGG ATG AC-3′; 5′-GCC AGG ACA CTT GGG ATG AC-3′; IL-10: 5′-TGT GAA AAT AAG AGC AAG GCA GTG-3′; 5′-CAT TCA TGG CCT TGT AGA CAC C-3′; TGF-β: 5′-GAC CCT GCC CCT ATA TTT GGA-3′; 5′-CGC CCG GGT TGT GTT G-3′. The relative RNA expression of a gene of interest is presented relative to the endogenous control (β-actin) as 2^−ΔCt^.

### 2.2. In Silico Studies

#### 2.2.1. Analysis of the Expression Levels of H_2_S-Producing Enzymes in Human CD4+ T Cell Subset

In order to profile the expression pattern of the three hydrogen sulfide (H_2_S)-producing enzymes (i.e., CTH, CBS, and MPST) in human CD4+ T cell subsets, we interrogated the GSE43005 microarray dataset, obtained from the Gene Expression Omnibus (GEO) databank [48]. The use of whole-genome expression databases has been largely used to identify novel pathogenetic pathways and therapeutic targets for disorders, such as autoimmune diseases and cancer [49,50,51,52,53]. Whole-genome transcriptomic data from primary naïve CD4+ T cells, as well as CD4+ T central memory cells, Th1, Th2, Th17 and Treg cells from four healthy people were included in the GSE43005 dataset [54]. The analysis was performed on the preprocessed and normalized gene expression matrix, as supplied by the original authors [54].

#### 2.2.2. Analysis of the Expression Levels of H_2_S-Producing Enzymes in PBMCs from MS Patients

To determine the transcriptomic levels of the H_2_S-producing enzymes in PBMCs from MS patients in both relapsing and stable disease, as well as in healthy donors, we interrogated the GSE138064 microarray dataset [55], downloaded from the GEO database. The dataset included data from 10 drug-naïve Relapse-Remitting (RR) patients in stable disease (age 45.2 ± 2.6) and 9 patients undergoing a clinical relapse (age 46.3 ± 3.5), along with 8 healthy donors (age 42.3 ± 4.8) [55]. The women to men ratio was 8/2 and 8/1 for the stable and relapsing patients, respectively, and 5/3 for the healthy donors. All the MS patients were diagnosed with clinically definite or laboratory-supported disease, with an Expanded Disability Status Scale (EDSS) of 0–6.5. The patients had no concurrent infections, nor had they used glucocorticoids in at least the six months prior to the study. Complete clinical data and sampling procedures are available in Feng et al., 2019 [55]. The analysis was performed on the preprocessed and normalized gene expression matrix, as supplied by the original authors [55].

#### 2.2.3. Computational Deconvolution of Immune Cells Population

A computational deconvolution analysis was performed to infer the relative proportion of immune subpopulations within the PBMCs from the MS patients and healthy donors. The web-based software, xCell (http://xcell.ucsf.edu/ (accessed on January, 2020), was used [56], as it allows to determine in a sample, by using its transcriptomc signature, the enrichment of several cell types, including active dendritic cells (aDCs), astrocytes, B cells, CD4+ naive T cells, conventional DCs (cDCs), memory B cells, plasma cells, Th1 cells, Th2 and Treg cells and monocytes/macrophages. Cell type enrichment and correlation were visualized as heatmaps, generated using the xCellView web-based utility (https://comphealth.ucsf.edu/app/xcellview/) [56].

### 2.3. Statistical Analysis

One-way ANOVA followed by Tukey’s multiple comparison test or hte Student’s t-test (two-tailed) were used as appropriate for the statistical analysis using GraphPad Prism version 6.00 for Windows, (GraphPad Software, La Jolla, CA, USA). A *p* value less than 0.05 was considered statistically significant. Data of the in silico analyses are shown as the mean ± SD of log2 expression values and the statistical analysis was performed using the linear model for microarray (LIMMA) algorithm. A Benjamini–Hochberg adjusted *p* value (false discovery rate, FDR) <0.05 was considered as threshold for statistical significance. Statistical analysis was performed using GraphPad Prism 8 (La Jolla, CA, USA). and MeV (v. 4.9; Rockville, Maryland, USA) softwares.

## 3. Results

### 3.1. In Vitro, In Vivo and Ex Vivo Studies

#### 3.1.1. Effects of GYY4137 on Dendritic Cells

**Figure 1:**
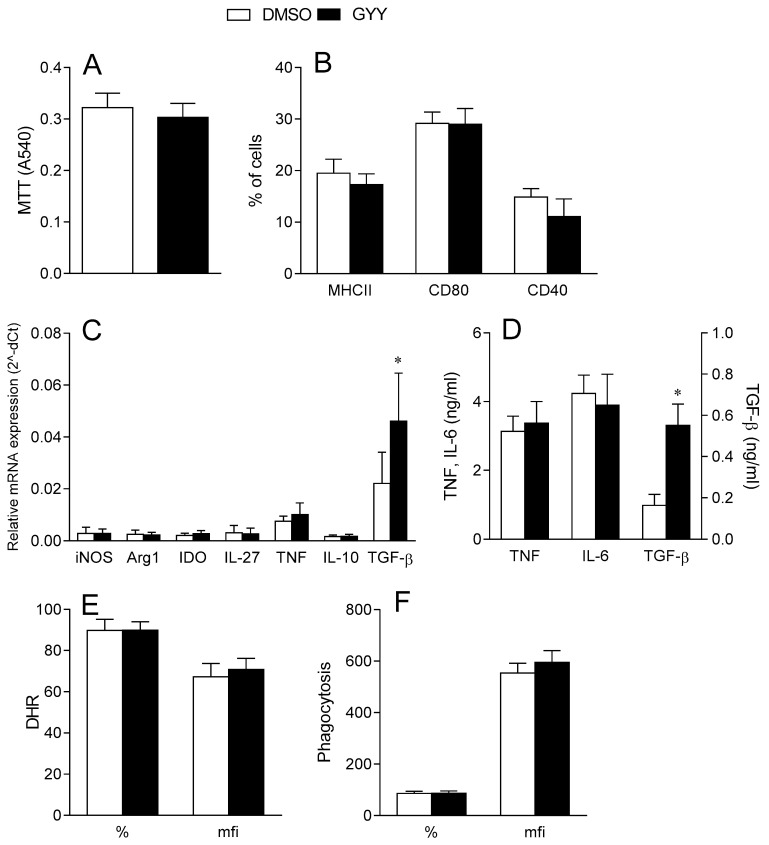
Effects of GYY4137 on bone marrow-derived dendritic cells (BMDCs). BMDCs were propagated from mouse bone marrow precursors in the presence of Granulocyte-macrophage colony-stimulating factor (GM-CSF) and matured under the influence of lipopolysaccharide (LPS). Moreover, 200 μM GYY4137 (GYY) was applied simultaneously with LPS and DMSO was used as the vehicle control (DMSO). The cell viability was determined by the MTT test (**A**), the expression of MHC II, CD80 and CD40 was determined by cytofluorimetry (**B**), the mRNA expression relative to β-actin was detected by real-time RT-PCR (**C**), the cytokine concentrations were determined by ELISA (**D**), phagocytosis was determined by cytofluorimetry (**E**), and reactive oxygen species (ROS) production was measured by DHR staining and cytofluorimetry (**F**). Data are presented as the mean + standard deviation (SD) from three (**F**), four (**B**), five (**A**,**C**,**E**) or six (**D**) samples. * *p* < 0.05 refers to DMSO.

Murine BMDCs were differentiated in vitro in the presence of GM-CSF and matured under the influence of LPS. GYY4137 (200 M) was applied simultaneously with LPS for 24 h. The dose of 200 M was chosen as it was previously shown to be efficient in modulating the phenotypic and functional properties of microglial cells [47] without affecting cell viability. Indeed, at this dose, GYY4137 did not affect the DC viability (Figure 1A). Moreover, GYY4137 had no effect on the expression of MHC class II molecules, the CD80 and CD40 co-receptors (Figure 1B), the mRNA levels of various cytokines (Figure 1C), pro-inflammatory cytokines TNF and IL-6 release (Figure 1D), reactive oxygen species generation (Figure 1E) and phagocytosis (Figure 1F). On the contrary, both the TGF-β mRNA level as well as the TGF-β release were higher in the BMDCs in GYY4137-treated cultures in comparison to solvent (DMSO)-treated cultures (Figure 1C,D).

#### 3.1.2. Effects of GYY4137 on Mouse Lymph Node T cells

**Figure 2:**
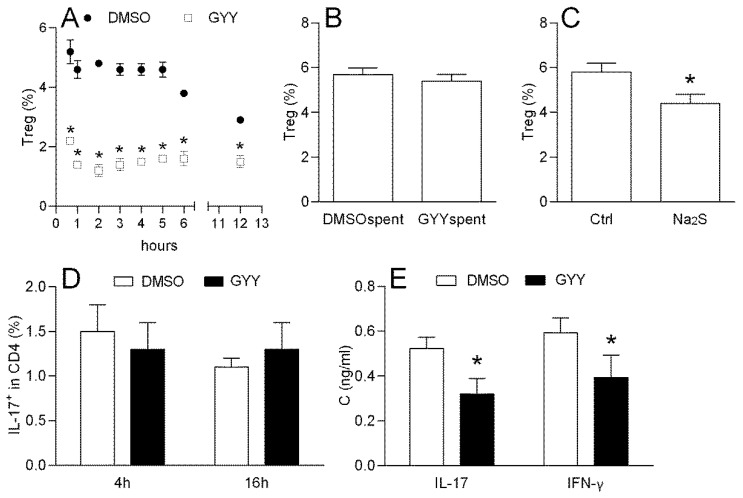
Effects of GYY4137 on the mouse draining lymph node cells (DLNCs). The DLNCs were isolated from the mice immunized with myeline oligodendrocyte glycoprotein (MOG) + complete Freund’s adjuvant (CFA). The percentage of CD4+ CD25+ FoxP3+ cells (regulatory T cells (Treg)), and the proportion of IL17+ among the CD4+ cells were determined by cytofluorimetry. The percentage of Treg among DLNCs was measured after different time points of treatment with 200 μM GYY4137 (GYY) or DMSO as the solvent control (DMSO) (**A**). The percentage of Treg among the DLNCs was measured after 40 min of treatment with 200 μM of spent GYY4137 (GYYspent) or spent DMSO as the solvent control (DMSOspent) (**B**), or with Na2S 200 μM or no treatment (Ctrl) (**C**). Percentage of IL-17+ cells among CD4+ cells was determined among the DLNCs, treated with GYY4137 (GYY) or DMSO as the vehicle (DMSO) for 40 min and subsequently stimulated for 4 h or 16 h (**D**). IL-17 and IFN-γ cytokine concentrations in the supernatants of 24 h cell cultures of MOG re-stimulated DLNCs treated with GYY4137 were determined by ELISA (**E**). The data are presented as the mean + standard deviation (SD) from three (**A**–**C**) or six (**D**,**E**) samples. * *p* < 0.05 refers to DMSO (**A**,**B**,**D**,**E**) or Ctrl (**C**).

DLNCs obtained from mice immunized with MOG + CFA were treated with GYY4137 (200 M) from 40 min to 12 h and the percentage of CD4+CD25+FoxP3+ cells (Treg) was determined by flow cytometry. The treatment led to the sustained reduction in the proportion of Treg cells among the DLNCs obtained from mice (Figure 2A). GYY4137 that was kept in the cell culturing medium for 7 days at 37 °C in order to completely release H_2_S (spent GYY4137) did not have any effect on the Treg proportion (Figure 2B), thus suggesting that H_2_S, and not some other product of its decomposition, was responsible for the observed effect. This was consistent with the fact that the effect on the Treg proportion was mimicked by an alternative H_2_S donor—Na_2_S (Figure 2C). DLNCs were also treated with GYY4137 for 40 min and stimulated with PMA and Ionomycine in the presence of Brefeldin A for 4 or 16 h. The percentage of CD4+IL-17+ (Th17) cells among the DLNCs was not altered under the influence of GYY4137 (Figure 2D). However, the IL-17 and IFN-γ levels were decreased in the supernatants of 24 h cell cultures of DLNC re-stimulated with MOG and treated with GYY4137 (Figure 2E).

#### 3.1.3. Effects of GYY4137 on the Rat Lymph Node T Cells

**Figure 3:**
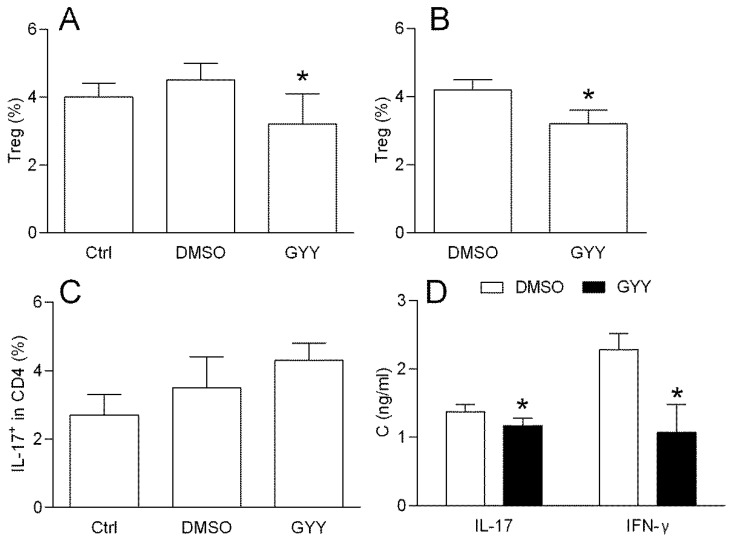
Effects of GYY4137 on the rat DLNCs. The DLNCs were isolated from the rats immunized with MOG + CFA. The percentage of CD4+ CD25+ FoxP3+ cells (Treg), and the proportion of IL17+ among the CD4+ cells were determined by cytofluorimetry. The percentage of Treg was determined among the DLNCs (**A**) or among CD4+ T cells purified from the DLNCs (**B**), after 40 min of cultivation in the presence of 200 μM GYY4137 (GYY) or DMSO as the vehicle (DMSO). The percentage of Th17 cells was determined among the DLNCs, treated with GYY4137 (GYY) or DMSO as the vehicle (DMSO) for 40 min and subsequently stimulated for 4 h (**C**). In some experiments, the cultures without DMSO (Ctrl) were also performed (**A**,**C**). IL-17 and IFN-γ cytokine concentrations in the supernatants of the 24 h cell cultures of MOG re-stimulated DLNCs and treated with GYY4137, were determined by ELISA (**D**). Data are presented as the mean + standard deviation (SD) from three (**C**), four (**B**) or six samples (**A**,**D**). * *p* < 0.05 refers to DMSO.

DLNCs obtained from the rats immunized with MOG + CFA were treated with GYY4137 (200 μM) for 40 min and the percentage of CD4+CD25+FoxP3+ cells (Treg) was determined by flow cytometry. The treatment led to the reduction in the proportion of Treg cells among the DLNCs (Figure 3A). The reduction of the Treg proportion under the influence of GYY4137 was also detected in the CD4+ cells purified with rat DLNCs (Figure 3B), thus implying that the donor had direct effects on the CD4+ T cells. Alternatively, the cells were treated with GYY4137 for 40 min and stimulated with PMA and Ionomycine in the presence of Brefeldin A for 4 h. The percentage of IL-17+ cells among the DLNC CD4+ T cells (Th17) was not altered under the influence of GYY4137 (Figure 3C). However, the IL-17 and IFN-β levels were decreased in the supernatants of 24 h cell cultures of DLNCs re-stimulated with MOG and treated with GYY4137 (Figure 3D).

#### 3.1.4. Effects of GYY4137 on Spinal Cord T Cells

**Figure 4:**
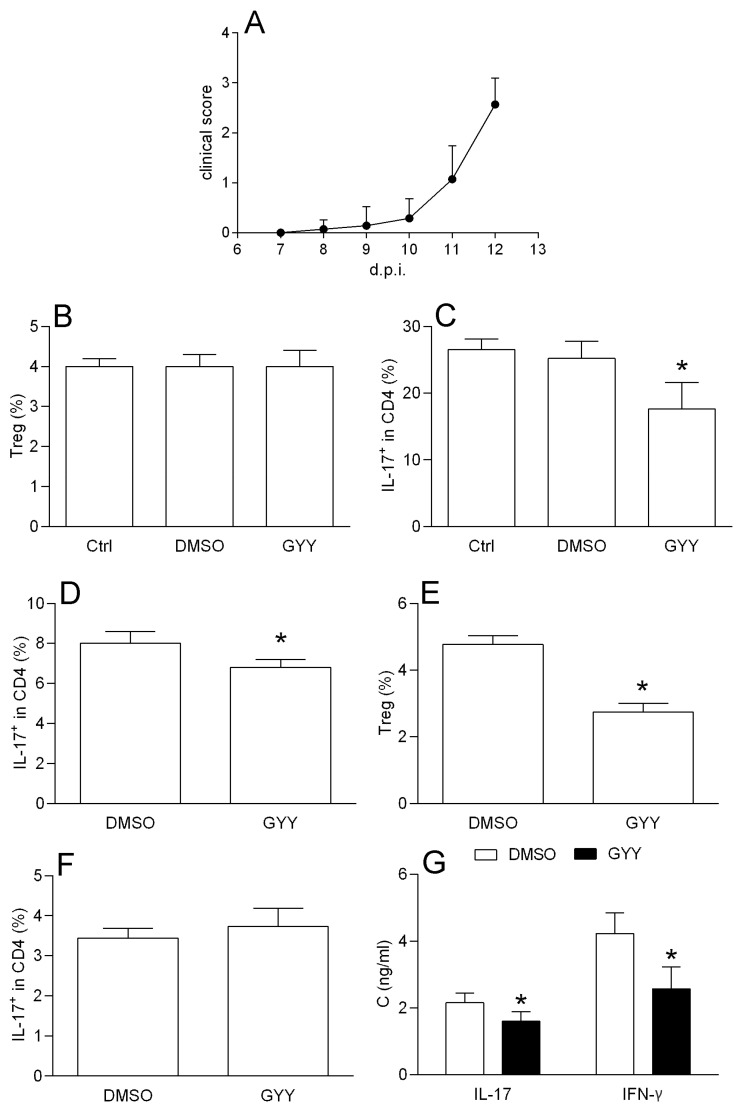
Effects of GYY4137 on rat spinal cord immune cells (SCICs). SCICs and DLNCs were isolated from rats immunized with rat spinal cord homogenate (SCH) + CFA at the peak of the disease or at day 7 post immunization, respectively. Clinical course of the EAE is presented (**A**). The percentage of the CD4+ CD25+ FoxP3+ cells (Treg), and the proportion of IL17+ among the CD4+ cells were determined by cytofluorimetry. The percentage of Treg was determined among the SCICs after 40 min cultivation in the presence of 200 μM GYY4137 (GYY) or DMSO as the vehicle (DMSO) (**B**) The percentage of IL-17+ cells among the CD4+ cells was determined among the SCICs (**C**) or among the CD4+ T cells purified from the SCICs (**D**), after 40 min cultivation in the presence of 200 μM GYY4137 (GYY) or DMSO as the vehicle (DMSO). The percentage of Treg was determined among the DLNCs after 40 min cultivation in the presence of 200 μM GYY4137 (GYY) or DMSO as the vehicle (DMSO) (**E**). The percentage of IL-17+ cells among the CD4+ cells was determined among the DLNCs (**F**) after 40 min cultivation in the presence of 200 μM GYY4137 (GYY) or DMSO as the vehicle (DMSO). In some experiments, the cultures without DMSO (Ctrl) were also performed (**B**,**C**). IL-17 and IFN-γ cytokine concentrations in the supernatants of 24 h cell cultures of SCICs treated with GYY4137 were determined by ELISA (**G**). Data are presented as the mean + standard deviation (SD) from three (**C**,**D**,**F**), four (**B**,**E**) or six (**A**,**G**) samples. * *p* < 0.05 refers to Ctrl.

SCICs were obtained from rats immunized with SCH + CFA at the time of the EAE peak. The treatment with SCICs with GYY4137 for 40 min had no effect on the Treg proportion (Figure 4A). However, the proportion of Th17 was reduced after the GYY4137 treatment, both in complete SCICs, and in CD4+ T cells purified from SCICs (Figure 4B,C). Importantly, GYY4137 reduced Treg, but not the Th17 proportion among the DLNCs obtained from the SCH + CFA immunized rats (Figure 4D,E), thus excluding the possibility that the observed difference between the data obtained with the SCICs and the DLNCs was the consequence of a discrepancy in the immunization. Finally, the GYY4137 reduced the levels of IL-17 and IFN-γ in SCIC culture supernatants in comparison to the cultures treated with DMSO (Figure 4F).

### 3.2. In Silico Studies

#### 3.2.1. Expression of H_2_S-Producing Enzymes in Human CD4+ T Cell Subsets

**Figure 5:**
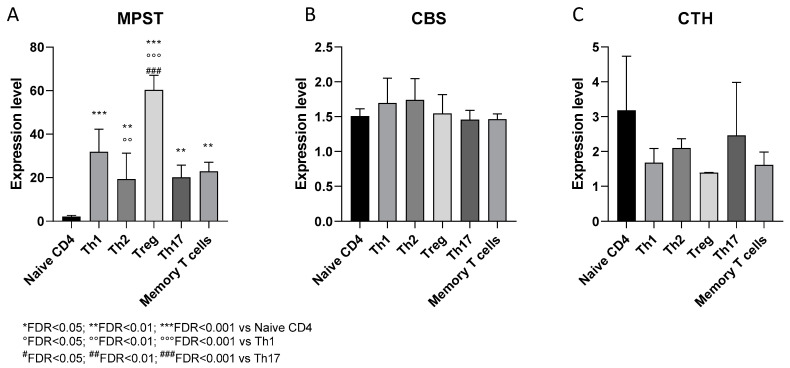
Expressions of the H_2_S-producing enzymes in human CD4+ T cell subsets. The expression levels of Mercaptopyruvate Sulfurtransferase (MPST) (**A**), Cystathionine Beta-Synthase (CBS) (**B**), and Cystathionine Gamma-Lyase (CTH) (**C**) were evaluated in human CD4+ T cell obtained from the the peripheral blood mononuclear cells (PBMCs) of healthy donors, by interrogating the publicly available GSE43005 microarray dataset, retrieved from the the Gene Expression Omnibus (GEO) database (https://www.ncbi.nlm.nih.gov/gds) [48].

The relative expression levels of the three hydrogen sulfide (H_2_S)-producing enzymes, MPST, CBS and CTH, were investigated in human CD4+ T cell subsets, namely naïve CD4+ T cells, Th1, Th2, Tregs, Th17 and memory T cells. Significantly higher levels of MPST were observed in all the subsets of polarized T helper cells, compared to the naïve CD4+ T cells (FDR < 0.01) (Figure 5A). Moreover, the Treg cells expressed significantly higher levels of MPST compared to both Th1 and Th17 cells (FDR < 0.01) (Figure 5A). No significant alteration was instead observed in the expression levels of the CBS and CTH for any T helper cell subset (Figure 5B,C).

#### 3.2.2. Expression of H_2_S-Producing Enzymes in PBMCs from Multiple Sclerosis Patients

**Figure 6:**
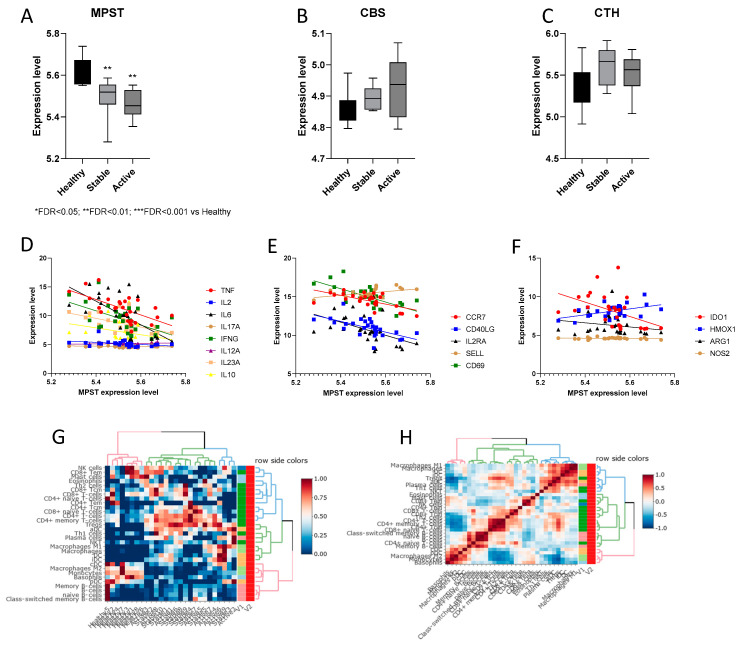
Expression of the H_2_S-producing enzymes in th ePBMCs from multiple sclerosis (MS) patients. The expression levels of Mercaptopyruvate Sulfurtransferase (MPST) (**A**), Cystathionine Beta-Synthase (CBS) (**B**), and Cystathionine Gamma-Lyase (CTH) (**C**) were evaluated in the PBMCs from healthy donors and drug-naïve relapse-remitting MS patients, by interrogating the publicly available GSE138064 dataset, retrieved from the GEO database (https://www.ncbi.nlm.nih.gov/gds [48]). Data are shown in log2 scale. (**D**) Correlation between the MPST expression and the cytokines, the immune-activation markers (**E**) and the anti-inflammatory enzymes expression (**F**), as determined in the GSE138064 dataset. The deconvolution analysis of the immune cells in the PBMCs from healthy donors and MS patients, as determined in the GSE138064 dataset, is presented as the heatmap of the cell enrichment (**G**) and correlation (**H**) analysis.* FDR < 0.05; ** FDR < 0.01; *** FDR < 0.001.

Significant lower expression levels of MPST were determined in the PBMCs from the MS patients in active and relapsing disease, as compared to the healthy donor PBMCs (FDR < 0.01) (Figure 6A). No significant differences in the MPST expression were instead found in the PBMCs from patients in relapsing disease as compared to the PBMCs from stable MS patients (Figure 6A). On the other hand, no difference could be observed for the CBS and CTH between the healthy donor and MS samples (Figure 6B,C). A correlation analysis revealed that the MPST expression inversely correlated with the pro-inflammatory cytokines TNF (*p* < 0.001), IL6 (*p* < 0.001), IFNG (*p* < 0.01) and IL23A (*p* < 0.01) (Figure 6D, Table 1). A significant inverse correlation was also observed between the MPST and CCR7 (*p* < 0.001), CD40L (*p* < 0.001), IL2RA (CD25) (*p* < 0.05), and CD69 (*p* < 0.0001) (Figure 6E, Table 1), while a significant positive correlation was found between MPST and SELL (CD62L) (*p* < 0.05) (Figure 6E, Table 1). Moreover, the expression levels of MPST were inversely correlated to IDO1 (*p* < 0.05) and positively correlated with HMOX1 (*p* < 0.05) (Figure 6F, Table 1). In order to evaluate whether the differences in MPST expression were associated to changes in the cell composition of MS patients compared to healthy donors, a deconvolution analysis was performed. The cellular composition was strikingly different between the healthy individuals and the multiple sclerosis patients, while no separation could be observed between the patients in stable and active disease (Figure 6G). A higher proportion of both Th1 and Treg was found in the MS patients, along with an increased percentage of memory CD4+ T cells and M1 macrophages (Figure 6G,H).

## 4. Discussion

We showed here, for the first time, that the H_2_S donor GYY4137 exerts different effects in vitro and ex vivo on immune cells relevant for EAE pathogenesis. Having a limited effect on BMDCs, GYY4137 reduces the Treg cells originating from lymph nodes, but not from the CNS. Moreover, the donor diminishes the ability of T cells to generate IL-17 and IFN-γ. Furthermore, our in silico analysis of H_2_S-producing enzyme expression revealeda an elevated MPST expression in the Tregs of healthy donors, as well as the reduced MPST gene expression in the PBMCs from multiple sclerosis patients. However, no differences in the CBS and CTS gene expression were determined.

The role of H_2_S in the CNS inflammation has gained increasing attention in recent years [11,57]. The therapeutic potential and neuroprotective effects of H_2_S were demonstrated in several CNS diseases, including Alzheimer’s disease, Parkinson’s disease, ischemic stroke, Huntington’s disease, amyotrophic lateral sclerosis and traumatic brain injury [57,58]. Regarding MS, the anti-inflammatory, anti-apoptotic and anti-oxidant effects of H_2_S in CNS [57] are highly relevant. Specifically, the protective effects of H_2_S in neurons and astrocytes that have been reported [59,60] suggest that this molecule could be beneficial in MS. Along the same line, we and others have previously shown that H_2_S potently affects the inflammatory activity of microglia [39,47]. However, the effect of H_2_S on encephalytogenic cells during MS and EAE has not been addressed.

In this study, GYY4137 did not affect DCs viability, the expression of MHC class II molecules, CD80 and CD40 co-receptors involved in T cell activation, the mRNA levels of various cytokines, phagocytosis, reactive oxygen species generation or the release of the pro-inflammatory cytokines TNF and IL-6. However, it markedly up-regulated TGF-β mRNA levels and the release of this cytokine in these cells. TGF-β is a pleiotropic cytokine that supports the differentiation and function of CD4+ Treg, which are important in limiting the autoimmune response in MS and EAE [61]. The observed up-regulation of TGF-β mRNA levels in DCs suggests that the anti-inflammatory effect of GYY4137 on DCs is highly relevant for MS. It is an immunopharmacological objective to keep DC TGF-β production higher than IL-6 so to shift balance of CD4+ T cell differentiation from Th17 to Treg [62]. It would be important to investigate if the effects of GYY4137 on DC TGF-β production are relevant for support to Treg differentiation. As the first step, in vitro study of Treg differentiation from naïve T cells in the presence of GYY4137-treated and un-treated DCs should be performed.

The proportion of Treg was not affected while that of Th17 was reduced when GYY4137 was applied to the immune cells isolated from the inflamed CNS, and the production/release of IL-17 and IFN-γ was also reduced. This implies that H_2_S may affect re-stimulated T cells in the target tissue. Importantly, the inhibitory effects of GYY4137 on Th17 cells were obtained in purified CD4+ cells. This suggests that the effects of H_2_S on T cells are direct, rather than indirect through other immune cells present in the isolates, such as macrophages/microglia. The inhibitory effects of H_2_S on IL-17 generation in the CNS have been previously reported [63], thus corroborating our findings.

In our study, GYY4137 reduced the proportion of Treg both in rat and mouse CD4+ T cells from draining lymph nodes after 40 min treatment, having no effect on the percentage of Th17 cells. Importantly, another H_2_S donor Na_2_S, had a statistically significant yet limited effect on Treg proportion. This discrepancy in the intensity of the effect could be a consequence of different kinetics of the donors. While GYY4137 releases H_2_S with a peaking time of approximately 10 min and sustains H_2_S release for over an hour, Na_2_S releases H_2_S in seconds [36]. In accordance with the relatively slow release of H_2_S from GYY4137, its effect on Treg proportion was sustained for more than 12 h.

Importantly, GYY4137 had different effects on the CD4+ T cells obtained from the CNS and from the periphery. This may be secondary to the fact that the cells are differently conditioned in vivo, and therefore respond differently to GYY4137 ex vivo. Indeed, T cells isolated from the spinal cord at the time of the EAE peak may mimic in vivo reactivation, that would not occur in the T cells obtained from the DLNCs during the inductive phase of the disease [64,65], and these differences can make them vary in their molecular signature [66,67]. Furthermore, the Treg from DLN differ from those obtained from the CNS in their autoantigen affinity and methylation status of Treg-specific demethylated region [68]. It has been suggested that most of the CNS, but not the DLN Treg, are of thymic origin in EAE [68]. It will be important to decipher the molecular mechanisms of H_2_S effects on Treg and Th17 and to discover why T cells isolated from the CNS and from the periphery respond to GYY4137 differently. This is especially important if GYY4137 and other H_2_S donors are considered for therapeutic application in MS. Thorough spatial- and kinetics-related molecular analysis of the effects of H_2_S on Treg and Th cells in the CNS autoimmunity is mandatory to fully understand the feasibility of H_2_S tailored approaches for the prevention and treatment of these diseases.

A significant reduction in the MPST expression was found in the PBMCs from the MS patients compared to the PBMCs from healthy donors. This result was particularly important in light of our finding that the slow-releasing donor of H_2_S has an inhibitory effect on the production of pro-inflammatory cytokines in encephalitogenic cells. Thus, the down-regulated expression of MPST in the PBMCs of MS patients may imply that impaired H_2_S production contributes to the pathogenesis of MS. Inverse correlation between MPST expression and the expression of different molecules with pro-inflammatory properties (including pro-inflammatory cytokines, CD40L, CD69, IL2RA, CCR7) is consistent with the anti-inflammatory function of H_2_S. Furthermore, we have previously shown a reduced HMOX1 expression in the PBMCs of multiple sclerosis patients [15]. Therefore, a positive correlation between the MPST and the HMOX1 expression in PBMCs strengthens the concept of a beneficial role of this H_2_S-producing enzyme in MS. As the ability of cells expressing IDO1 genes to suppress immune responses is well known [69], we studied the correlation between MPST and IDO1 expression. The negative correlation confirmed in our study indicates that the elevated MPST expression may not necessarily be accompanied by immuno-suppression or immune tolerance or that IDO1 independent mechanisms are activated by H_2_S. It is in line with the fact that the effects of H_2_S on immune cells are divergent and dependent on multiple factors.

The increasing evidence indicating an important role for H_2_S in the modulation of immune responses makes the expression of H_2_S-producing enzymes in immune cells important targets for the better understanding of the role of this gas in the pathogenesis of MS. Except for the association of mutant CBS gene variants with an earlier onset of MS [12], to date, H_2_S-producing enzymes have not been examined in the context of MS. In silico analysis performed in our study revealed higher MPST expression levels in Treg as compared to the Th1 and Th17 cells of healthy donors. This is in line with the importance of cysteine for Treg suppressive activity [70] that entails a competitive uptake of cysteine, during the activation of T cells. While cysteine is preferentially catabolized to sulfate in Treg, in effector T cells cysteine is preferentially utilized for protein synthesis. As MPST is a part of the cysteine catabolism pathway, the higher expression of this enzyme in Treg potentially contributes to reducing the cysteine availability during the activation of T cells. Moreover, the production of H_2_S as the result of elevated MPST expression might represent an additional mechanism of suppression by Treg. However, because several studies suggest that most of the H_2_S produced by MPST is stored in the form of sulfane sulfure, the significance of this potential mechanism of suppression needs additional studies [37]. Although this could explain the up-regulated MPST expression in Treg compared to Th1 and Th17 cells, it does not clarify why MPST expression is substantially lower in naïve CD4+ T cells compared to all the subsets of polarized T helper cells in healthy individuals. Together with other cysteine catabolic pathways, the mercaptopyruvate pathway contributes to pyruvate production, which can be further oxidized and used in the tricarboxylic acid cycle [71]. It is thus possible that a lower MPST expression could be a reflection of metabolic quiescence of naïve CD4+ T cells [72].

Although no differences in the CBS and CTH gene expression were observed among the analyzed samples, there could still be variances in their enzymatic activity. Both the CBS and CTH can also be regulated at the posttranscriptional level. While the H_2_S-producing activity of CTH is also dependent on intracellular Ca^2+^ concentrations [73], CBS activity is regulated by a heme cofactor which serves as a redox sensor, or via the posttranslational regulation through S-glutathionylation [74,75]. Thus, the comparison of CBS and CTH activity in the different T cell populations obtained from healthy individuals and MS patients is worthy of studies.

## 5. Conclusions

Our results imply that H_2_S predominantly exhibits an anti-inflammatory effect on the T cells involved in a prototypic model of experimental CNS autoimmunity in EAE. Further in vitro studies on the molecular pharmacological mechanisms of GYY4137 effects, as well as its in vivo application in EAE, are required to assess the potential of specific H_2_S donors in the treatment of MS and possibly other autoimmune diseases. The potential clinical relevance of our findings is highlighted by the fact that H_2_S-releasing drugs, such as SG1002 for cardiovascular disorders, and ATB-346 for arthritis, have progressed into clinical trials and have shown considerable promise [76]. Hence, the emerging demonstration of the defective synthesis/function of H_2_S in some cases of MS along with the immunomodulatory effects of H_2_S donation in encephalytogenic cells in vitro could propel immediate biomarker-driven clinical studies with H_2_S donors in MS.

## Figures and Tables

**Table 1:** Correlation between the MPST expression and the markers of immune activation as determined in the GSE138064 dataset.

	Pearson r	95% Confidence Interval	R Squared	*p* Value
MPST vs. TNF	−0.6234	−0.8112 to −0.3189	0.3886	0.0005
MPST vs. IL2	−0.3639	−0.6536 to 0.01865	0.1325	0.0620
MPST vs. IL6	−0.6283	−0.8140 to −0.3261	0.3947	0.0004
MPST vs. IL17A	−0.1955	−0.5357 to 0.1993	0.0382	0.3285
MPST vs. IFNG	−0.548	−0.7681 to −0.2122	0.3003	0.0031
MPST vs. IL12A	0.3633	−0.01940 to 0.6531	0.1320	0.0625
MPST vs. IL23A	−0.492	−0.7347 to −0.1378	0.2421	0.0091
MPST vs. IL10	−0.3544	−0.6472 to 0.02964	0.1256	0.0697
MPST vs. CCR7	−0.6298	−0.8148 to −0.3284	0.3967	0.0004
MPST vs. CD40LG	−0.6226	−0.8108 to −0.3177	0.3876	0.0005
MPST vs. IL2RA	−0.4755	−0.7246 to −0.1166	0.2261	0.0122
MPST vs. SELL	0.4422	0.07476 to 0.7039	0.1956	0.0209
MPST vs. CD69	−0.6878	−0.8465 to −0.4167	0.4730	<0.0001
MPST vs. IDO1	−0.4533	−0.7109 to −0.08856	0.2055	0.0176
MPST vs. HMOX1	0.4613	0.09860 to 0.7158	0.2128	0.0154
MPST vs. ARG1	−0.2147	−0.5498 to 0.1800	0.0461	0.2822
MPST vs. NOS2	−0.1639	−0.5120 to 0.2305	0.0269	0.4140

## Supplementary Materials

The following are available online at https://www.mdpi.com/2076-3921/9/7/608/s1, Figure S1: Treg gating strategy, Figure S2: Th17 gating stretegy.
